# Impact of N221S missense mutation in human ribonucleotide reductase small subunit b on mitochondrial DNA depletion syndrome

**DOI:** 10.1038/s41598-023-47284-5

**Published:** 2023-11-14

**Authors:** Leila Su, Xin Wang, Jianghai Wang, Frank Luh, Yun Yen

**Affiliations:** 1 Sino-American Cancer Foundation, Covina, CA 91722 USA; 2 Ph.D. Program for Cancer Biology and Drug Discovery, College of Medical Science and Technology, Taipei Medical University, 250 Wu‐Hsing Street, Taipei, 110301 Taiwan; 3 Center for Cancer Translational Research, Tzu Chi University, Hualien, 970374 Taiwan

**Keywords:** Biochemistry, Computational biology and bioinformatics, Structural biology, Diseases, Medical research

## Abstract

The impact of N221S mutation in hRRM2B gene, which encodes the small subunit of human ribonucleotide reductase (RNR), on RNR activity and the pathogenesis of mitochondrial DNA depletion syndrome (MDDS) was investigated. Our results demonstrate that N221 mutations significantly reduce RNR activity, suggesting its role in the development of MDDS. We proposed an allosteric regulation pathway involving a chain of three phenylalanine residues on the αE helix of RNR small subunit β. This pathway connects the C-terminal loop of β2, transfers the activation signal from the large catalytic subunit α to β active site, and controls access of oxygen for radical generation. N221 is near this pathway and likely plays a role in regulating RNR activity. Mutagenesis studies on residues involved in the phenylalanine chain and the regulation pathway were conducted to confirm our proposed mechanism. We also performed molecular dynamic simulation and protein contact network analysis to support our findings. This study sheds new light on RNR small subunit regulation and provides insight on the pathogenesis of MDDS.

## Introduction

Mitochondria are semi-autonomous organelles in mammalian cells that contain their own independent genome, mitochondrial DNA (mtDNA). They continuously and independently replicate mtDNA to produce energy and metabolic intermediates required for cellular activities^1,2^. Mitochondrial functions are regulated by both nuclear and mitochondrial DNA. Mutations in either genome can lead to heterogenous mitochondrial impairment, resulting in mitochondrial genetic diseases such as mtDNA depletion syndromes (MDDS) and mtDNA deletion disorders^3,4^. Human RRM2B (hRRM2B) mutations are known to cause infantile-onset MDDS^5,6^. Penque et al. reported a case study of a newborn with a novel homozygous missense mutation, N221S, in hRRM2B^7^. The newborn developed signs and symptoms consistent with MDDS and ultimately died at 3 months of age. Structural analysis of hRRM2B found that N221 forms a polar-π interaction with the highly conserved F206 residue close to the active site^7^. It was proposed that the shorter side chain introduced by the N221S mutation increases the distance between S221 and F206, disrupting the interaction between the two residues and negatively impacting RNR mtDNA regulation^7^.

Ribonucleotide reductase (RNR) is the only enzyme in living cells that converts ribonucleotides to deoxyribonucleotides for DNA synthesis and repair^8^. Class I RNR is a heterotetrametric complex (α2β2) consisting of two dimer subunits, α (RRM1) and β (RRM2 and RRM2B). α is the larger and catalytic subunit responsible for reducing ribonucleotides, while β is the smaller subunit for radical generation. Mammalian cells have two forms of β subunit^9^: RRM2, regulated by the cell cycle and expressed mainly during the S phase for de novo dNTP synthesis; and RRM2B (also known as p53R2), regulated by the p53 tumor suppressor protein. RRM2B is continuously expressed at low levels throughout the cell-cycle for DNA repair and maintenance of dNTP pools for mtDNA synthesis in non-proliferative cells^9,10^. Balance and control of the mitochondrial deoxynucleotide pools are essential for the maintenance of mtDNA integrity and copy number. Perturbation of this homeostatic control can lead to multi-organ failure, developmental regression, failure to thrive, and even neonatal death^5,11,12^.

The small subunit β of class Ia RNR contains a unique eight-helix bundle (α-barrel) core with very long helices. In *E. coli* RRM2, αE helix consists of 39 residues and αB helix has 32 residues^13^, in contrast to typical α-helices^14^. A binuclear iron center (Fe1Fe2) with a strictly conserved tyrosine residue (e.g., Y122 in *E. coli* RRM2) nearby is sandwiched between αB and αE helices (Figure 2a). β has two hydrophobic oxygen-binding pockets, each located at oxygen or water binding site of the binuclear iron center^13^. Our previous work identified a cross protein pore in hRRM2B extending all the way to the Fe2 site^15^. This pore provides increased accessibility to the iron and oxygen binding sites in hRRM2B and suggests a possible gating mechanism for oxidation regulation. When the inactive RNR complex is bound by ATP, RRM1 (α) dimerizes and binds to RRM2 (β) dimer to form an active RNR heterotetramer (α2β2) complex^8,16^. The activation signal from the ATP-bound RRM1 reaches RRM2 via C-terminal loop (C-loop) of RRM2, where a tyrosyl radical (e.g., Y122 in *E. coli* RRM2) is produced at the active site by oxidation^17^. The radical is then transported to RRM1 for ribonucleotide reduction via a long-range proton-coupled electron transfer (PCET) pathway^18,19^. In the cryo-EM holocomplex structure of *E. coli* RNR active form, the C-loop of RRM2 forms a β-sheet that pairs with β-sheets from RRM1, wraps around RRM1 with Y356 at the interface, and lands at the catalytic site of RRM1^20^. Once a deoxynucleotide product is formed and ATP is dephosphorylated, the C-loop disassembles from RRM1 and returns to a random structure state, thereby reverting RNR to its inactive form.

In this study, we investigated the molecular mechanisms underlying the disease-causing N221S mutation in the context of MDDS and aimed to elucidate the regulation of radical activation and transportation in RNR small subunit β. We proposed an allosteric regulation pathway in β that transfers the activation signal from the C-loop to the αE helix and then to the radical generation site via a phenylalanine chain, which consists of three phenylalanine residues arranged in an orderly fashion along the same face on the unusually long αE helix. One of the phenylalanine residues (F206 in hRRM2B) interacts with a critical asparagine residue (N221 in hRRM2B), which holds the αE helix in place. We evaluated the effects of mutations in residues involved in the phenylalanine chain and the regulation pathway using in vitro site-directed mutagenesis studies and computational perturbation analyses to confirm our hypothesis. In addition, molecular dynamic simulations and protein contact network analysis were performed to further validate the proposal.

## Results

### hRRM2B mutagenesis study

The fatal consequences of N221S mutation^7^ led to a thorough investigation of the impacts of N221 side chain, a conserved residue in RNR small subunit. Site-directed mutagenesis studies involving six N221 constructs were performed to elucidate the molecular mechanism of N221S related MDDS. The mutations were designed to introduce different side chains with varying properties, such as hydrophilic (N221Q) or hydrophobic (N221V and N221L), smaller (N221S and N221T) or larger in size (N221Q and N221Y), as well as hydroxyl groups (N221S, N221T, and N221Y), to explore their impact on enzyme activity and stability. In addition to the N221 mutants, mutations were also made to residues (F198, F202, F206, K210, and F311) that are involved in the N221 contact network, the phenylalanine chain, or the regulation pathway identified by computational and structural analysis.

**Figure 1:**
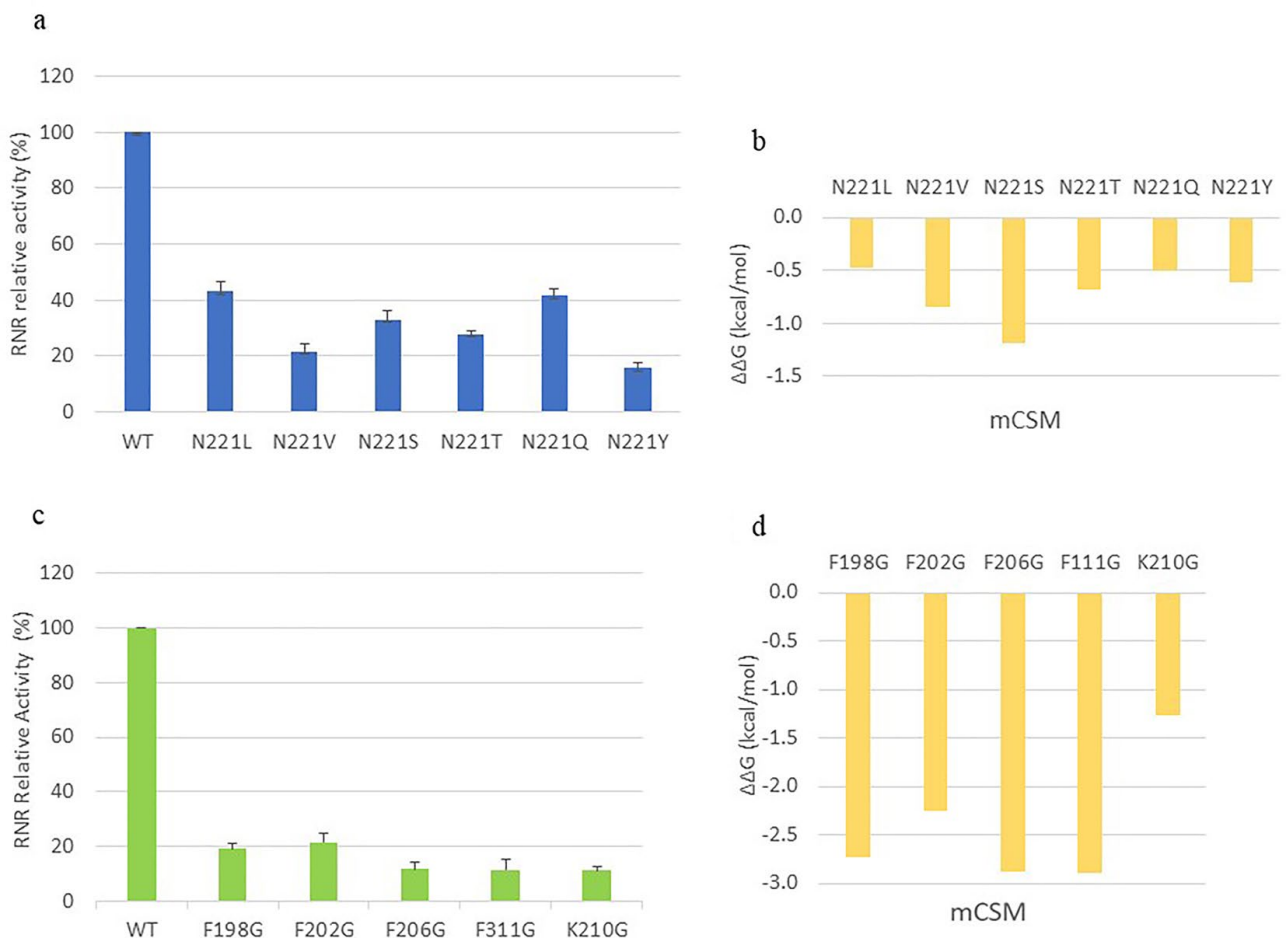
Experimental and computational mutagenesis studies of hRRM2B. (**a**) Experimental data of N221 mutations. (**b**) Computational mutagenesis data of N221 mutations using mCSM algorithm. Negative ΔΔG values indicate unfavorable substitutions. (**c**) Experimental data of F198G, F202G, F206G, F311G, and K210G mutations. (**d**) Computational calculation of F198G, F202G, F206G, F311G, and K210G mutations by mCSM. The same quantity of recombinantly purified wild-type or mutant hRRM2B were used in each assay for the comparison. The reactivity was reflected by reduction of CDP to dCDP of RNR complex. Amount of dCDP formed was quantified by HPLC peak area and normalized with the wild-type value. Relative RNR activities of hRRM2B mutants in complex with RRM1 are used to evaluate mutation effects. Each column is a mean value from three independent experiments, and the error bars represent the range of each data set.

All the designed mutations and the wild-type hRRM2B were expressed and purified to homogeneity, and confirmed by SDS-PAGE analysis (Figure S1a). The enzymatic activity of the RNR complex in converting CDP to dCDP was determined using an RNR activity assay, with final concentrations of hRRM1 and hRRM2B (wild-type or mutants) at 2 μM and 6 μM, respectively. The results showed that all N221 mutants exhibited a significant reduction in enzymatic activity, ranging from 57 to 85% of the wild-type value (Fig. 1a). This indicates that any changes to the N221 side chain can have a critical impact on RNR function, highlighting the importance of N221 for RNR functions. Mutations of F198, F202, F206, F311, and K210 also resulted in strong defect in RNR activity, ranging from 78 to 89% reduction of the wild-type value (Fig. 1c), thus confirming the functional importance of these residues. Circular dichroism studies were performed to ensure that protein folding in RRM2B mutants was still intact (Figure S1b), and that decrease in enzyme activity caused by mutant construct was not due to loss of protein structure. The retained activity of the mutants indicated that the mutant hRRM2Bs formed normal active complex with hRRM1. The data obtained from our mutagenesis studies, along with MDDS-related mutations from the literature involving N221 and the phenylalanine chain in different species, are summarized in Table 1.

**Table 1:** List of mutations from laboratories and MDDS in human (RRM2 and RRM2B) and *E. coli* RRM2.

hRRM2B		hRRM2		*E. coli* RRM2	
Residue	Activity (%)	Residue	Activity (%)	Residue	Activity (%)
F198G	**19.1**	F236A	9.2**ǂ**	F208Y*	0.0
F202G†	**21.5**	F240A	3.1**ǂ**	F212Y* F212W*	0.0 0.0
F206G	**11.7**	F244G	**73.2**	F216	NA
N221S‡	**33.0**	N259	NA	I231	NA

*NA* not available.

The unpublished results are highlighted as bold.

Enzyme activity determined in Yen’s lab was presented as percentage of dCDP formation of wild type protein.

The specific activity of hRRM2 and hRRM2B is 4008 and 5710 (nmol/min/mg), respectively (See ‘Methods’ section).

Each value is the average of three determinations with deviations < 0.3

The references for mutagenesis data and MDDS mutations are listed as *^33^ ǂ^26^ †^22^ ‡^7^.

Computational simulations were conducted to assess the effect of the mutations on protein stability using the mutational Cutoff Scanning Matrix (mCSM) machine learning algorithm^21^. The algorithm calculated the free energy change (∆∆G) of each mutant, which was used to evaluate the effect of the mutations on protein stability. Negative ΔΔG values indicate that the substitutions are unfavorable and could destabilize the protein (Fig. 1b,d). Consistent with our experimental assays, all alternations to N221 side chain destabilized the protein (Fig. 1b). Of all the N221 mutants, N221S was found to be the most unstable mutant according to mCSM analysis. This destabilization was attributed to the removal of the NH2CO functional group (N221L & N221V), changes in the size of the N221 side chain (N221Q & N221T), and the addition of a hydroxyl group reductant, suggesting all three factors contribute to the pathogenicity of N221S mutation. The mCSM algorithm also demonstrated higher destabilization effects by all the phenylalanine mutations (Fig. 1d), which is consistent with our comparative RNR enzymatic studies. Interestingly, mCSM calculation suggested that K210G mutant is less destabilizing than the phenylalanine residues, which is different from the enzyme activity study. The differences between the computational simulation and the experimental data (Fig. 1a,b,c,d) suggest that mCSM algorithm can accurately predict the effects of a mutation at certain condition, considering many factors involved in experimental assays. On the other hand, the discrepancy could also suggest other interactions are involved with the residue. Since this is beyond the scope of the current study, we will not elaborate further.

### Importance of the alpha *E. helix* in hRRM2B

**Figure 2:**
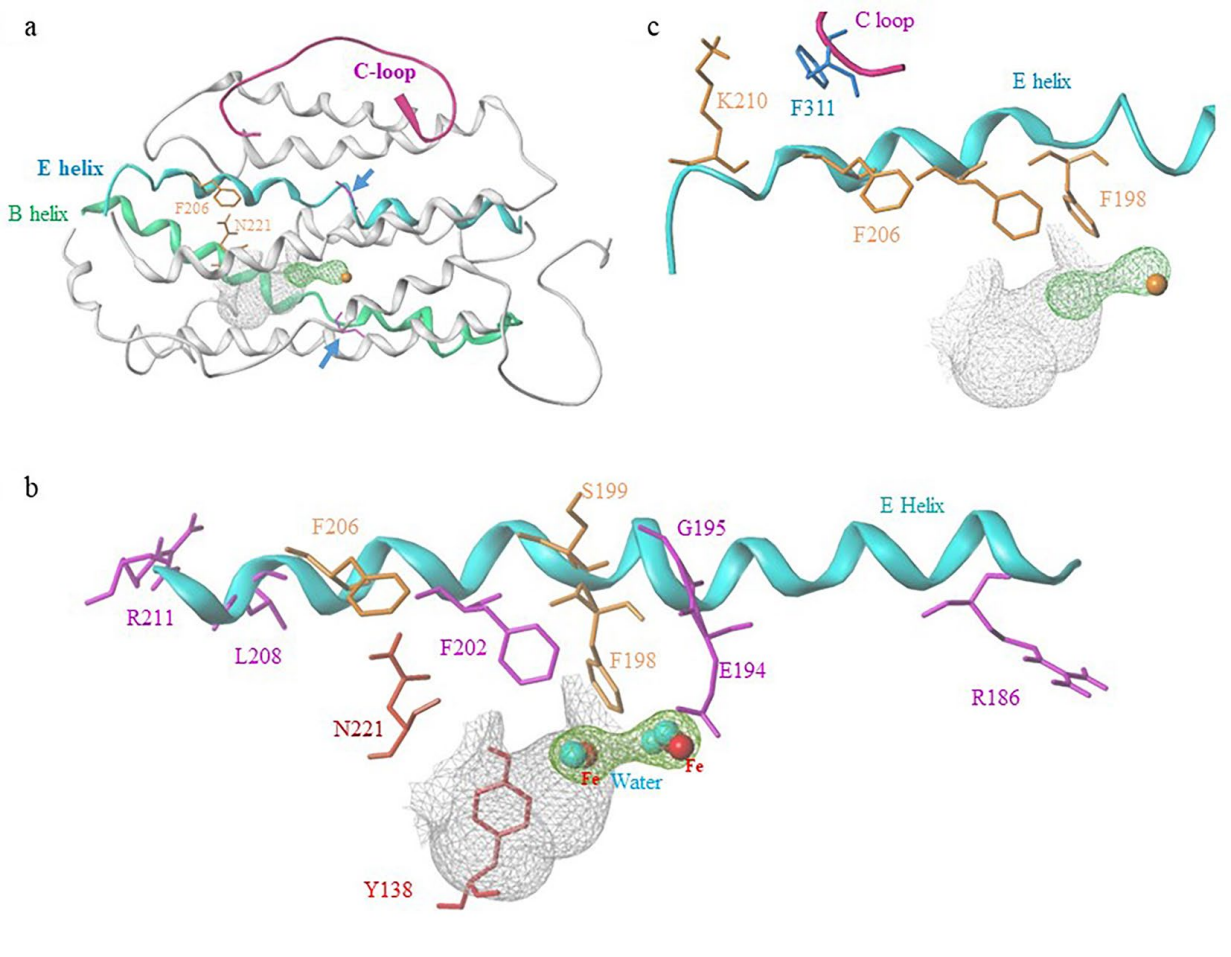
Structural analyses of hRRM2B. (**a**) The overall structure of hRRM2B (PDB ID 3HF1) depicted as gray ribbons. αB and αE helices, and C-terminal loop are highlighted in green, cyan, and magenta, respectively. Distortions of the two helices by the extra glycine (magenta) at the center of each helix are indicated by arrows. N221 and F206 are shown in orange. (**b**) Selected αE helix residues and N221, Y138. The six residues whose mutations are MDDS associated are colored in magenta. The other residues (F198, S199, F206) are shown in orange. N221 and Y138 are depicted in red. (**c**) The phenylalanine chain on αE helix: F198, F202, and F206. K210 and F311 pair are also shown. F311 is colored cyan and the others in orange. The water molecules are colored as atom types in space-filled rendering. The surface of active site and oxygen binding pocket are drawn in white and green, respectively. Protein structures were prepared using SYBYL.

In a previous study, we showed that F206 interacts with N221 in hRRM2B^7^. In this work, structural analysis revealed that F206 is located on the unusually long αE helix, which is situated between the C-loop and αB helix (Fig. 2a). The fact that the αE helix borders both the radical-iron center and oxygen-binding sites underscores its critical role in RNR function. The αB and αE helices both exhibit distortions near the iron center due to an amino acid residue in the middle of the helix (G101 in αB and G195 in αE of hRRM2B) in higher organisms (serine in *E. coli*). These well-conserved glycines confer flexibility in the region, opening the helices to enable assembly of the radical-iron cofactor and allowing molecular oxygen or water to reach the metal sites.

The significance of the αE helix is marked by known mutations associated with MDDS located on the αE helix of hRRM2B (Fig. 2b). One of the mutations, G195R, is a missense mutation of the helix breaker G195, and is pathogenic for autosomal dominant progressive external ophthalmoplegia (adPEO)^22^. Additionally, six other missense mutations (R186G, E194G, E194K, F202L, L208X, R211K) have been reported^12,23–25^. Three of these mutations (R186G, L208X, R211K) are situated at the ends of the αE helix (Fig. 2a), which suggests their involvement in intramolecular or intermolecular interactions. The other three mutations (E194G, E194K, F202L) are in the central region of the helix, near the iron/tyrosyl radical/oxygen binding pockets, indicating the indispensable role of these residues in enzyme function.

### Three important phenylalanine residues F198, F202, and F206 on the alpha *E. helix*

**Figure 3:**
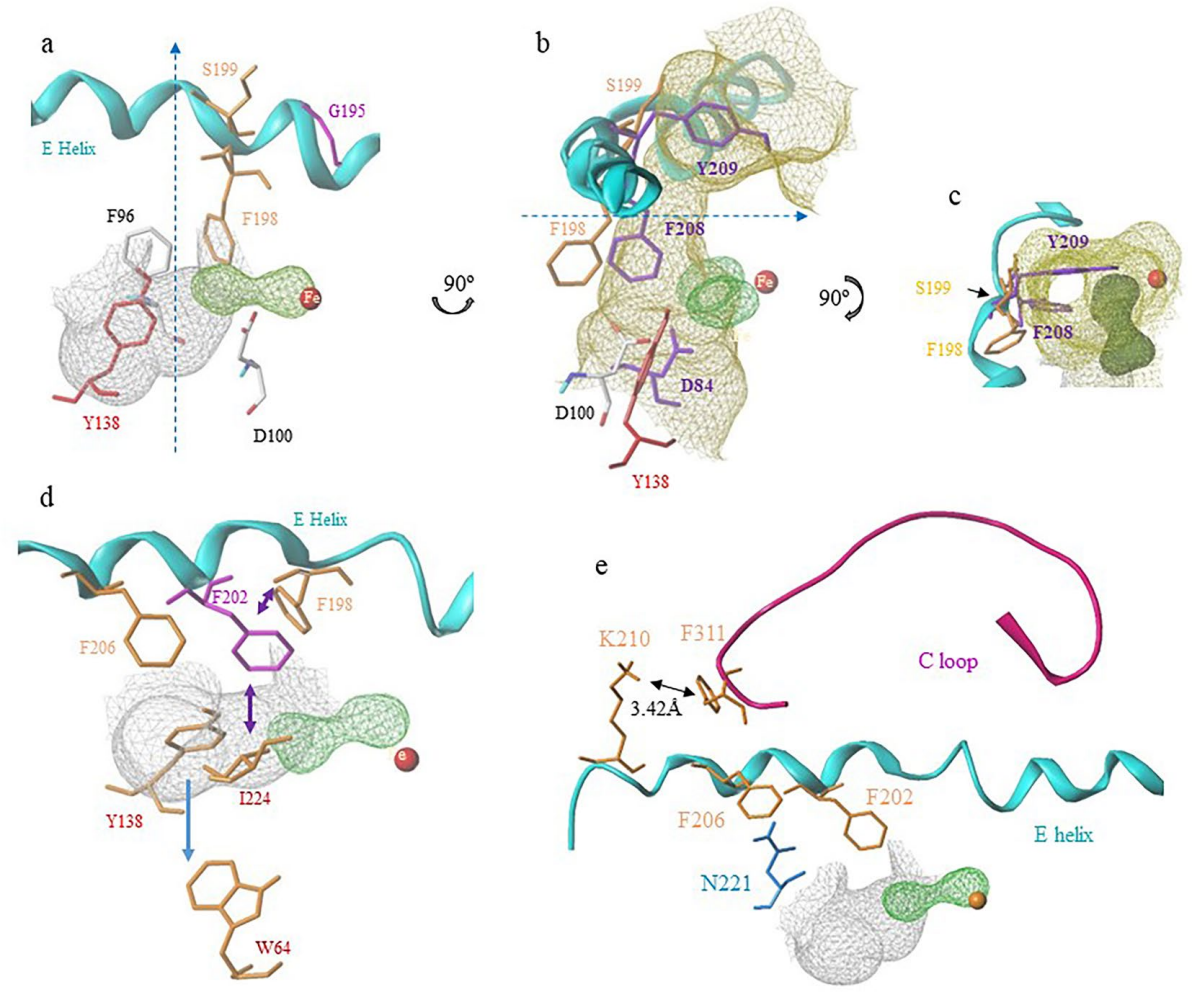
F198, F202, F206 of the phenylalanine chain. (**a**) F198 and S199 (both in orange), and G195 (magenta) are shown. F96 and D100 are colored as atom types. Y138 is colored red. (**b**) View of (**a**) rotated ninety degrees around an axis in the plane of the figure overlapping with residues from *E. coli* RRM2 (purple, PDB ID 6W4X). Some residues are omitted for clarity. An open channel is shown in yellowish green surface. (**c**) Bird’s eye view of (**b**) rotated ninety degrees around an axis shown in the figure. The hRRM2B open channel is shown with *E. coli* RRM2 residues F208 and Y209 overlaid to occlude the channel opening. (**d**) F202 is depicted in magenta. The other residues are in orange. The two-way arrows represent inter-residue interaction, and the one-way arrow indicates PCET pathway. (**e**) F206, F202, K210, and F311 are shown in orange. N221 is colored cyan. C-terminal loop of hRRM2B is shown in magenta. The distance between K210 and F311 is indicated in the figure. The other color codes of the structure features are shown as in Fig. 2.

Our structural analysis revealed important roles of three phenylalanine residues, F198, F202, and F206 on the αE helix of hRRM2B. F198 is involved in forming hydrophobic oxygen-binding pockets at the Fe1 and Fe2 water-binding sites (Fig. 2b). Mutation of F198 (F198G) led to an 80% reduction in hRRM2B activity (Fig. 1c). The corresponding residue in hRRM2 (F236) also exhibited a drastic reduction in activity with the mutant F236A retained only 9.2% of the wild-type hRRM2 activity^26^ (Table 1). Computational protein stability analysis confirmed the importance of F198 in RNR (Fig. 1d). Interestingly, when we compared the structure of hRRM2B with that of *E. coli* RRM2, we found that the analogous residue F208 in *E. coli* is located much closer to the oxygen-binding site than F198 in hRRM2B (Fig. 3b). In hRRM2B, F198 moves away from the oxygen-binding site, creating a pore that allows for increased accessibility to the iron and oxygen sites^15^. When residue S199 in hRRM2B, which also contributes to the pore formation, is substituted with Y209 in *E. coli*, the resulting Y209 blocks the oxygen channel (Fig. 3b,c). This Y-to-S switch, together with the conformational changes of the residue F208-*E. coli*/F198-hRRM2B, leads to an opening of the oxygen channel and increased accessibility to the iron site in hRRM2B. As shown in Fig. 3c, F208 and Y209 in *E. coli* RRM2 are positioned to occlude the pore, while F198 and S199 in hRRM2B move away to open the pore. Our findings suggest that this phenylalanine residue (F198 in hRRM2B, F236 in hRRM2, F208 in *E. coli*) acts as a conformational gate to regulate oxygen accessibility in small subunit β.

In hRRM2B, the hydroxyl group (OH) of the radical-forming tyrosine (Y138) is enclosed within a conserved hydrophobic pocket formed by F198, F202, N221, and I224 (Fig. 2b and 3d), which ensures the stability of the tyrosyl radical. Notably, F202, a strictly conserved residue, sits near both the radical site and the oxygen-binding pockets in hRRM2B (Fig. 3d). Our site-directed mutagenesis experiments targeting F202 in hRRM2B (Fig. 1c) and F240 in hRRM2 (Table 1) revealed that substituting the sidechains significantly affect RNR activity (F240A activity was 3.1% of the wild-type)^26^. F202L is a missense mutation associated with MDDS^26^ (Fig. 2b). Computational simulations further support the critical roles of F202 in stabilizing these crucial sites (Fig. 1d). Taken together, our study suggests that F202 plays several crucial role in maintaining the stability of the hydroxyl group of Y138 and the integrity of the oxygen-binding pockets.

Our structural analysis found that F206 in hRRM2B stabilizes the α-barrel core structure by interacting with surrounding residues, including N221 of the αF helix (Fig. 3e). This interaction between the phenylalanine-asparagine pair in hRRM2B results in an optimal side chain interaction where N221 stacks on top of the aromatic ring of F206 for a polar-π interaction. Our mutagenesis study and computational simulation on F206 in hRRM2B showed significant effects from the F206 mutation, confirming our view that F206 is critical in stabilizing the protein core and interaction network (Fig. 1c, d).

### The phenylalanine chain is part of RNR allosteric regulation pathway

The three phenylalanine residues (F198, F202, and F206) in hRRM2B separated by four amino acids each are orderly arranged on the same face of the αE helix to form a phenylalanine chain. This chain interacts with the PCET pathway and could play a key role in enzyme regulation (Figure S2). F198 is in close contact with D100 in hRRM2B (Fig. 3a), and it is believed that D100 is involved in a conformation gating regulation, similar to the *E. coli* RRM2 PCET pathway^19^. In *E. coli,* regulation of radical formation occurs by a conformational gate involving the radical Y122-O^•^ and iron ligand D84 (equivalent to D100 in hRRM2B) at different redox states^19,27,28^. In this report, our findings are in line with our previous study, which found D100 in hRRM2B is flexible with multiple conformations^15^. In the previous study, one monomer of the hRRM2B dimer, D100 swings away from the active site; while in the other monomer, D100 reorients to bind iron, indicating an interplay between the inactive and active monomers in hRRM2B dimer and a conformational gating regulation involving D100^15^. In the current study, we propose that F198 is also involved in conformational gating regulation through D100 (Fig. 3a,b). Furthermore, F198 stacks with F96, which, in turn, interacts with Y138 (Fig. 3a). Thus, F198 likely stabilizes and regulates the Y138 tyrosyl radical via the stacking/interacting cascade of F198/F96/Y138 in hRRM2B.

Our structural analysis shows that F202 participates in a hydrophobic network connecting the radical generation site to the PCET pathway. F202 is located near I224 (Fig. 3d), a residue associated with MDDS^23^ that is in van der Waals (VDW) contact with both W64 and Y138, crucial residues for radical generation and transfer^19^. F202 also interacts with F198, forming an interaction network of W64-I224-F202-F198. Taken together, a regulatory signal near the protein surface close to W64 can be transmitted all the way to F198, which is in proximity to the buried radical/iron/oxygen sites (Fig. 3d). The crosstalk of this hydrophobic interaction network with the PCET pathway suggests that F202 may have a regulatory role in the enzyme.

**Figure 4:**
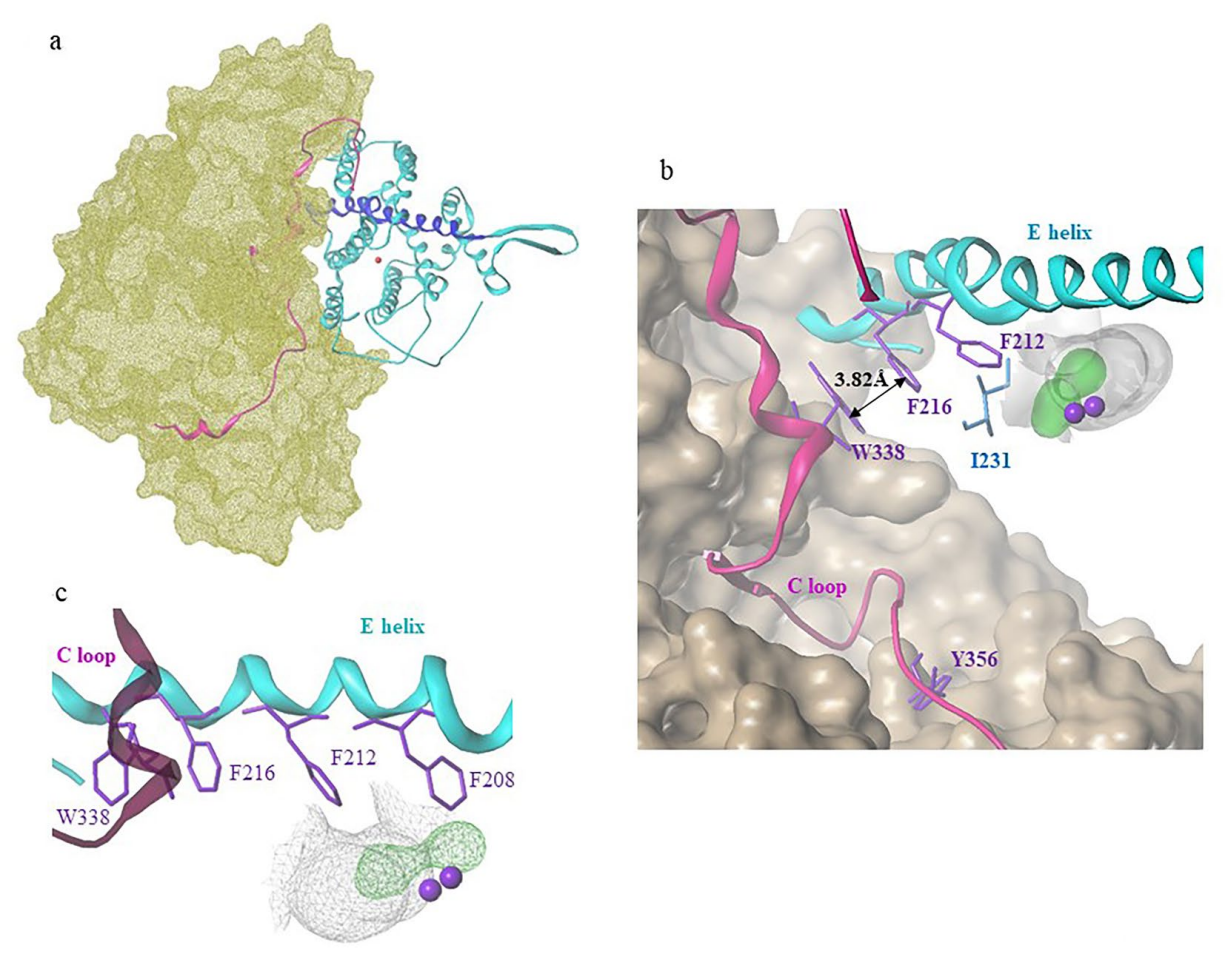
The allosteric regulation in RRM2 shown with *E. coli* RNR structure (PDB ID 6W4X). (**a**) The overall structure of *E. Coli* holocomplex (PDB ID 6W4X, chain B and C). RRM2 (chain C) is depicted as cyan ribbons imbedded in RRM1 (Chain B) surface (yellow). The C-loop and αE helix are highlighted in magenta and blue, respectively, with the C-loop wrapping around RRM1. Helices A, F, H, and part of E form the interface with RRM1. Fe2 of the diiron (Fe1Fe2) from the X-ray structure is shown and colored red. (**b**) Part of the enlarged RRM1 and RRM2 interface shown detailed interactions between αE helix and the C-loop. F216, F212, W338, and Y356 are shown in purple. I231 is colored in cyan. The distance between F216 and W338 is shown. (**c**) The phenylalanine chain in *E. coli* RRM2: F208, F212, and F216 on αE helix, with W338 on C-loop, are colored purple. The other color codes of the structure features are shown as in Fig. 2.

F206 proved to be an important residue in the regulation pathway. Specifically, in *E. coli*, the analogous residue F216 stacks with W338 of the C-loop (3.8 Å in distance, as shown in Fig. 4c) , which stabilizes the C-loop's position when RRM2 binds to the activated RRM1^20^. By comparison, F216 is further away from the analogous residue I231 in *E. coli* than F206 and N221 in hRRM2B. In *E. coli*, F216 is the outmost residue in the RRM2 core to contact the C-loop, which then forms β-sheets with RRM1 (Fig. 4b). This C-loop anchor, composed of F216/W338, is critical in ensuring that Y365, a restrict conserved residue involved in PCET, is correctly positioned between the two subunits (RRM1 and RRM2) for electron transfer. Instead, hRRM2B uses a lysine/phenylalanine pair of K210 on the αE helix and F311 on the C-loop to stabilize the C-loop (3.4 Å in distance, Fig. 3e). This interaction replaces the F216/W338 anchor used in *E. coli* and highlights different mechanisms used by these two organisms to achieve the same functional outcome.

We propose that when RRM1 is activated by ATP and forms a heterotetramer complex with RRM2, the C-loop of RRM2 interacts with the αE helix and transmits signals from RRM1 to RRM2, activating radical generation and PCET (Fig. 4a). This conformational change of the C-loop from a random coil to an ordered β-tail between the inactive and active states allosterically regulates signal transduction and RNR activity (Fig. 4b). Furthermore, when RRM2 is activated by binding to RRM1, the conformation of F198 near the oxygen binding site changes, unblocking a substrate channel and allowing oxygen access to the di-iron site in hRRM2B (Fig. 3c). Our findings support the notion that phenylalanine chain is involved with an allosteric regulation pathway that transmits signals from the C-loop to the RRM2 active site.

### *E. coli* cryo-EM structure analysis, MD simulation, and perturbation analysis

**Figure 5:**
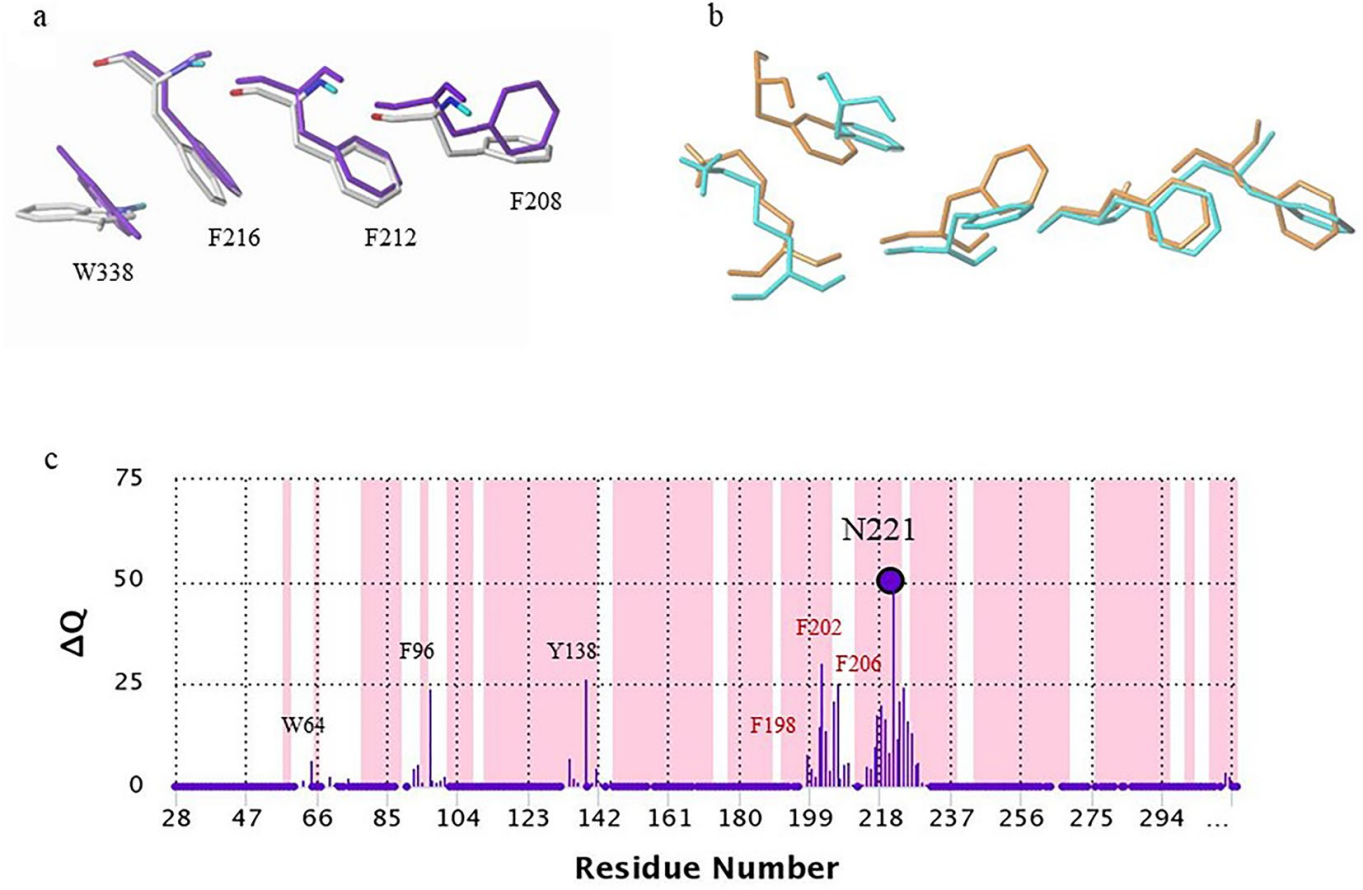
Regulation network in RRM2. (**a**) Comparison of the phenylalanine chain and the anchor point residues in the active and inactive states of *E. coli* RRM2 (PDB ID 6W4X, chains C and D, respective). Conformational changes of F208, F212, F216, and W338 between active (colored purple) and inactive (colored as atom types) are shown. (**b**) Comparison of the phenylalanine chain and anchor point residues of the last snapshot hRRM2B structure retrieved from the 500 ps production phase molecular dynamics simulation (orange) with the crystal structure (PDB ID 4DJN chain A, cyan). (**c**) Perturbation profile (∆Q vs. Cα—Cα) distance from the perturb site N221 (highlighted by a circle). The shaded regions represent the secondary structure elements in the input PDB file (magenta: α-helix). Some of the residues were labeled to visualize the predicted network of N221.

A comparison of the active and inactive *E. coli* RRM2 cryo-EM structures (PDB ID 6W4X, chains C and D, Fig. 5a) confirms the proposed regulatory role of the phenylalanine chain. In the active form (pre-turnover state, purple), the RNR complex and PCET pathway remain intact. F216 in the phenylalanine chain stacks with W338 from the C-loop, while F208 is in a position that opens the oxygen binding site. In the inactive form (post-turnover state, white), the C-loop of RRM2 dissociates from RRM1, and the side chain of W338 moves away from F216 (Fig. 5a). At the same time, F208 flips into its closed position, blocking oxygen uptake. This observation confirms that the C-loop residue W338 and the αE helix residue F208 act in a concerted way, supporting our hypothesis that the phenylalanine chain on the αE helix transmits radical activation signal from the RRM1 surface to the RRM2 active site via F216/W338 interaction.

To validate the allosteric regulation of the phenylalanine chain, we conducted molecular dynamic (MD) simulations on the wild type hRRM2B and N221S variant structures for 500 ps. The root-mean-square deviation (RMSD) of the hRRM2B wild type structure in the simulation trajectory is shown in Figure S3a. The RMSD value is 0.1 – 0.2 Å, indicating small structural deviations and stability during MD simulation. Comparison of the phenylalanine chain and anchor point residues of the last snapshot retrieved from MD simulation (Fig. 5b, in orange) with the hRRM2B wild type crystal structure (PDB ID 4DJN chain A, Fig. 5b, in cyan) showed that the conformational changes of these residues were like those found in *E. coli* RRM2 (Fig. 5a). The conformational features of the MD simulated structure (Fig. 5b, in orange) resembled the active (open) form of the *E. coli* enzyme (Fig. 5a, in purple), while the X-ray structural features (Fig. 5b, in cyan) resembled the relaxed inactive form of *E. coli* (Fig. 5a, in white). MD simulation of the N221S mutant showed that the close interaction between N221 and F206 (3.15 Å in distance) observed in the wildtype protein was lost in the simulated N221S mutant structure, where the distance between S221 to F206 was 7.06 Å (Figure S3b).

The effects of protein mutation can extend beyond the first shell (within 6 Å) from the mutated site and reach into the second shell (6 Å to 12 Å) from the site^29^. To investigate the impact of N221 and F206 mutation (mutated to alanine) to the interaction network of hRRM2B, we analyzed perturbation using the pPerturb^30^ server (Fig. 5c and Figure S3c). The perturbation magnitude (ΔQ) figure shows the degree of disturbance at each residue in the protein affected by the mutation. Perturbation analysis of N221 indicated that the phenylalanine chain residues (F198, F202, and F206), radical generation tyrosine Y138, radical stabilizing residue F96, and PCET residue W64 were all affected by the N221A mutation (Fig. 5c), consistent with our structural analysis (Fig. 2 and 3). Similarly, the perturbation of F206 mutation also affected N221, phenylalanine chain residues (F198, F202), as well as the F138-stacking F96 (Figure S3c, Fig. 3a,b). The perturbation also confirmed the interactions between the C-loop and the αE helix, as the mutation of F206, a residue on αE helix, perturbed the anchor point residue F311 on the C-loop. The perturbation analysis confirms the N221 interaction network and our hypothesis of allosteric regulation of the phenylalanine chain via the interaction network.

### N221S mutation perturbs the allosteric regulation and leads to MDDS

Both mutagenesis studies and computational calculations have demonstrated that the N221S missense mutation associated with MDDS reduced RNR activity compared to the wild-type protein (Fig. 1a,b). This reduction in activity is consistent with moderately decreased catalytic activity seen in autosomal-recessive mutations that typically cause early-onset MDDS cases^31^. The severe mtDNA depletion caused by the N221S mutation highlights the crucial role that RRM2B plays in the supply of dNTPs, especially for the replication of mtDNA.

Structural analysis reveals that N221 interacts with F206 of the phenylalanine chain, which is part of the allosteric regulation pathway in small subunit β. Our mutagenesis study revealed that a F206 mutation has similar effects on enzyme activity (Fig. 1c), providing support for the proposed interaction between N221 and F206 as demonstrated by the structural analysis. The N221S mutation, which introduces a shorter side chain, increases the distance between S221 and F206, disrupting the interaction between the two residues^7^ and as a result, negatively impacts the allosteric regulation pathway via the phenylalanine chain. Molecular dynamic simulation and protein contact network analysis also support the proposed allosteric regulation network and the role of N221S mutation. The pathogenic variant N221S alters hRRM2B protein structural properties leading to hRRM2B dysfunction and deficiency, which results in loss of ribonucleotide diphosphates reduction to dNDPs (mtDNA depletion).

## Discussion

Our study has identified a phenylalanine chain on the αE helix in Class Ia RNR as a novel allosteric regulator of enzyme activity. This phenylalanine chain interacts with hRRM2B residue N221, as shown in Figs. 2 and 3, and our mutagenesis study demonstrated that N221 mutants had low enzyme activity (Fig. 1a). This is consistent with Penque et al.’s 2019 clinical report^7^, which involved an infant diagnosed with MDDS due to the hRRM2B N221S mutation. Recently, a study involving zebrafish and morpholino oligomer (MO) knockdown technique confirmed that N221S variant is a loss-of-function mutation for hRRM2B^32^. The mutagenesis results from our study and publications, as well as MDDS-related mutations from the literature involving N221 and the phenylalanine chain in human RRM2 and RRM2B, *E. Coli* RRM2 are listed in Table 1. All mutations of the three phenylalanine residues significantly reduced enzyme activity. The F208 mutant, F208Y, in *E. coli* RRM2 was found to be enzymatically inactive in a previous study^33^. Similarly, mutations of the analogous residue F202 in hRRM2B, including F212Y and F212W, as well as the F240A variant in hRRM2, abolished enzyme activities, demonstrating the critical role of this phenylalanine residue (Table 1). In our study, the corresponding mutant F244G in hRRM2 was able to retain 73.2% of the wild-type enzyme activity. These results support the proposed allosteric regulation pathway involving the phenylalanine chain and N221 in hRRM2B. The reduction in RNR activity with mutations on these residues suggests that the phenylalanine chain may play a role in signal transduction to the radical generation site, which is necessary for proper RNR function.

From a molecular mechanism perspective on how N221S hRRM2B is linked to MDDS, we believe the mutation disrupts protein core stability and allosteric regulation by losing interaction with F206. The proposal is supported by our mutagenesis studies, MD simulation, and perturbation analysis. This novel regulatory feature of the phenylalanine chain sheds new light on the regulation of radical activation and the PCET pathway in Class Ia RNR and provides potential new target sites for developing therapeutic RNR inhibitors. We are encouraged to further explore the potential impact of N221S on RNR redox property, as documented by previous studies^34^. Further in vitro characterization of the phenylalanine residues is necessary to support the proposed interactions. Investigation on molecular mechanisms of MDDS involving other relevant hRRM2B mutations in the future are warranted. The fact that mutations on N221 and the phenylalanine chain can be linked to MDDS highlights the importance and conservation of this pathway for mitochondrial homeostasis. It would be interesting to investigate whether the mechanism of radical chemistry regulation with the phenylalanine chain in oxygen-dependent Class Ia small subunit β occurs in other RNRs like the oxygen-independent RNRs.

## Methods

### Protein structure preparation and analysis

X-ray crystal structure of RNR small subunit of human RRM2B (PDB ID 3HF1^15^) and cryo-EM structures of *E. coli* RRM2 (PDB ID 6W4X^20^) were downloaded from RCSB Protein Data Bank (PDB)^35^. Protein structure preparation, visualization, and analyses were carried out using SYBYL-X 2.1 (Tripos-Certara, Inc.). hRRM2B and N221S mutant structures were prepared for molecular dynamic study by SYBYL protein preparation wizard with following steps: removal of crystal water molecules and other substructures from protein structure, addition of polar hydrogens, and fix sidechains. Structural alignments for comparison were made using the module of Align Structure by Homology in SYBYL.

### Molecular dynamics simulation study

The prepared wildtype hRRM2B and mutant N221S structures were subjected to Molecular Dynamics (MD) simulation using the MDWeb^36^ web portal (https://mmb.irbbarcelona.org/MDWeb/). N221S mutation was made by mutate residue operation in MDWeb. GROMACS FULL MD setup was performed using AMBER-99SB ∗ force field. Simple Box Solvent Molecular Dynamics (NPT) were simulated using the following settings: time steps 2 fs, a constant temperature on the system was maintained at 300 K, total time 500 ps, output frequency 500 steps with total 500 snapshots. Water molecules and ions were removed from MD trajectories to obtained dry trajectory for further analysis. The root mean square deviation (RMSD), radius of gyration (Rg), B-factor fluctuations along the trajectory and per residue were downloaded and plotted to confirm the protein backbone stability. The converts trajectory to a set of PDB operation were performed to obtain structures along the time evolution trajectory for dynamic analysis.

### Protein stability analysis

Computational mutagenesis study was carried out using mCSM tool^21^ (http://biosig.unimelb.edu.au/mcsm/stability), which uses machine learning algorithm with statistical scoring functions as inputs. To analyze the effect of mutations of hRRM2B, free energy changes (∆∆G) were calculated. Based on these calculations, protein stability and effect of mutation were analyzed.

### Structure perturbation analysis

Interaction network of hRRM2B and its mutants was modeled using the pPerturb^30^ server (https://pbl.biotech.iitm.ac.in/pPerturb/pperturbn.html). Perturbation residue scanning (PRS) was performed. Amino acids N221 and F206 were mutated to alanine and perturbation profiles (∆Q vs. Calpha-Calpha) distance from the perturb site were generated. The perturbation effects were analyzed on the interaction network strength.

### Construction of expression plasmids for hRRM2B mutants

The plasmid used for wild-type hRRM2B was described in Shao et al., 2004^37^, which expresses a full-length hRRM2B with a 6X histidine-tag on its N-terminal. For this study, pairs of PCR primers were designed to alter the codon for residue N221 so that the plasmid would express the hRRM2B protein bearing a designed amino acid residue at position of N221 (see Supplementary Information for primer sequences). Site-directed mutagenesis was performed using an Agilent Quickchange II Site-Directed Mutagenesis Kit (Santa Clara, California). All constructed plasmids were sent to Genewiz (South Plainfield, New Jersey) to verify DNA sequence of mutation.

### Protein purification of recombinant hRRM1, hRRM2B and mutants

Expression and purification of recombinant hRRM1 and hRRM2B, as well as the hRRM2B mutants followed the procedure^37^ with modifications. *E. coli* strain BL21-Gold (DE3)-RIL harboring plasmid pET28a with insertion of coding sequence for human RRM1 was grew until reaching an optical density of 0.6–0.8. The protein expression was induced with 0.5 mM IPTG for 17 h at 15 °C. Grown bacteria were harvested and lysed with 50 mM NaH_2_PO_4_, pH 7.0 containing 500 mM NaCl, 1X BugBuster, 0.1% Triton X-100, and 20 mM imidazole. Protease inhibitors pill (Roche, Indianapolis, IN), 10 mM b-mercaptoethanol, 0.5 M PMSF, lysozyme (2 mg per gram of bacteria pellet), Benzonase (2.5 units/ul) were added into the lysis buffer right before use. After centrifugation at 35,000 × g for 30 min at 4 °C, the supernatant was loaded onto Ni–NTA resin for purification of hRRM1^37^. Fractions with the purified recombinant hRRM1 were pooled together, concentrated and buffer changed with Amicon Ultra-15 centrifugal filter unit (MilliporeSigma, Burlington, MA) and put into hRRM1 storage buffer containing 50 mM HEPES, pH7.6, 100 mM KCl, and 15 mM MgCl_2_. Glycerol and DTT were added to protein to reach a final concentration of 20% and 10 mM, respectively, before the protein solution was aliquoted and stored in -80 °C. The hRRM2B wild-type and mutants were purified following the same procedure^37^, except the induction of expression was achieved with 1 mM IPTG for 2.5 h. at 37 °C. Protein concentration was determined using BioRad Quick Start Bradford Protein Assay kit. A BioTek Synergy H1 Hybrid Reader with software Gen 5, 2.09 was used for protein concentration assay. Purity of proteins was checked by SDS-PAGE on Invitrogen 4–12% Bis–Tris Plus premade gel. SDS-PAGE gel image acquired by BioRad Chemi Doc Touch imaging system, with software 1.1.0.4. 2 μg of purified recombinant protein (hRRM2, hRRM2B, and hRRM2B mutants N221L, N221Q, N221S, N221T, N221V, N221Y, F198G, F202G, F206G, F311G, K210G) was loaded to each lane. Simply Blue Safe Stain (Invitrogen) was used for visualization.

### Circular dichroism (CD) study

CD spectra of hRRM2B and the mutants (0.4 mg/ml) in phosphate buffer (50 mM NaH2PO4, pH 7.0) were recorded with an AVIV Model 430 Circular Dichroism Spectrometer with software version 3.38. All measurements were carried out at 25 °C using a 1-mm path length quartz cuvette between wavelength 250 to 195 nm. The data pitch was 0.1 nm with a 1-nm bandwidth at a scan speed of 1.0 nm/s. Each spectrum shown represents the average of three scans. All CD data were expressed as the molar ellipticity, [θ], in units of degrees square centimeter per decimole.

### RNR activity assay

RNR activity assays were conducted according to a protocol described earlier^37^ with modifications. First, 2 mM of hRRM1 was mixed with 6 mM of hRRM2B, or the mutants in 40 ml. The mixture was incubated on ice for 30 min to reconstitute RNR complex. The reconstituted enzymes were then mixed with 20 ml of premix reagents. The final 60 ml mixture contains 50 mM HEPES, pH 7.6, 100 mM KCl, 4 mM magnesium acetate, 60 μM FeCl_3_, 5 mM DTT, 2 mM ATP, and 2 mM NADPH. Reaction was initiated by adding 20 ml of premix to 40 ml of reconstituted RNR. The solution was incubated at 37 °C for 20 min, and then heated to 95 °C and stayed for 3 min to terminate the reaction, followed by cooling down on ice. The sample was centrifuged twice and the combined supernatant was injected into a Synergi 4 μm Hydro-RP column (Phenomenex) attached to a Hewlett Packard 1100 series HPLC. Data was recorded with ChemStation B.03. The chromatograph was resolved with 50 mM NH_4_H_2_PO_4_, pH3.5, and the peaks of CDP and dCDP were quantified. The relative activities of hRRM2B mutants were calculated by their peak area as percentage of the wild-type protein.

### Equipment and settings

SDS-PAGE gel image acquired by BioRad Chemi Doc Touch imaging system, with software 1.1.0.4. Application setting: Coomassie Blue. A BioTek Synergy H1 Hybrid Reader with software Gen 5, 2.09 was used for protein concentration assay.

AVIV Model 430 Circular Dichroism Spectrometer with software v3.38 was used for CD spectra. All measurements were carried out at 25 °C using a 1-mm path length quartz cuvette between wavelength 250 to 195 nm. The data pitch was 0.1 nm with a 1-nm bandwidth at a scan speed of 1.0 nm/s.

Hewlett Packard 1100 series HPLC with a Synergi 4 μm Hydro-RP column (Phenomenex). Data was recorded with ChemStation B.03.

All the protein structure figures (Fig. 2, 3, 4, 5a,b, S2b, S3b) are generated using SYBYL-X 2.1 (Tripos-Certara, Inc.). Protein activity figures (Fig. 1a,c) are generated using Microsoft Excel 2016. Data were obtained from a Hewlett Packard 1100 series HPLC. Protein stability prediction figures (Fig. 1b,d) are generated using MS Excel 2016. Data were generated with mCSM algorithm (http://biosig.unimelb.edu.au/mcsm/stability). Protein structure perturbation analysis figures (Fig. 5c, S3c) are made with the online pPerturb server (https://pbl.biotech.iitm.ac.in/pPerturb/pperturbn.html). The MD RMSD figure (Figure S3a) is the data output from MD calculation using the web portal MDWeb (https://mmb.irbbarcelona.org/MDWeb/). Figure S4 is the original SDS-PAGE gel. Figure S1a is the cropped image to improve the clarity and conciseness of the presentation. The CD spectra were generated using an AVIV Model 430 Circular Dichroism Spectrometer.

### Supplementary Information

Supplementary Information.

## Data Availability

DNA and mRNA sequences were obtained from GenBank (https://www.ncbi.nlm.nih.gov/genbank/). The sequence ID are NM_001033.4 for hRRM1and NM_015713.4 for hRRM2B. Data supporting Fig. 1 and Table 1 are publicly available in the figshare repository, as part of this record: RRM2BmutantActivity (figshare.com).
