# Supplementary material for: Impact of N221S missense mutation in human ribonucleotide reductase small subunit b on mitochondrial DNA depletion syndrome
Source: Sci Rep. 2023 Nov 14;13:19899. doi: 10.1038/s41598-023-47284-5 (PMC10645729; doi:10.1038/s41598-023-47284-5)
Supplement: Supplementary file 1 — Supplementary Information. [file 41598_2023_47284_MOESM1_ESM.pdf]

Impact of N221S Missense Mutation in Human Ribonucleotide Reductase Small Subunit B on Mitochondrial DNA Depletion Syndrome

Leila Su[1], Xin Wang[1], Jianghai Wang[1], Frank Luh[1], Yun Yen[2,3*]

[1]*Sino-American Cancer Foundation, Covina, California 91722 USA;*

[2]*Ph.D. Program for Cancer Biology and Drug Discovery, Taipei Medical University, Taipei, 110301, Taiwan;*

[3]*Center for Cancer Translational Research, Tzu Chi University, Hualien, 970374, Taiwan.*

[*]To whom correspondence may be addressed. Dr. Yun Yen. College of Medical Science and Technology, Taipei Medical University, 250 Wu-Hsing Street, Taipei, 110, Taiwan. Telephone: 886-2-27322782 ext. 113; Email: yyen@tmu.edu.tw

This work was supported by the SACF Drug Discovery Research Fund and in association with TMU Research Center of Cancer Translational Medicine of the Higher Education Sprout Project and Taiwan Ministry of Education (MOE).

S1a S1b

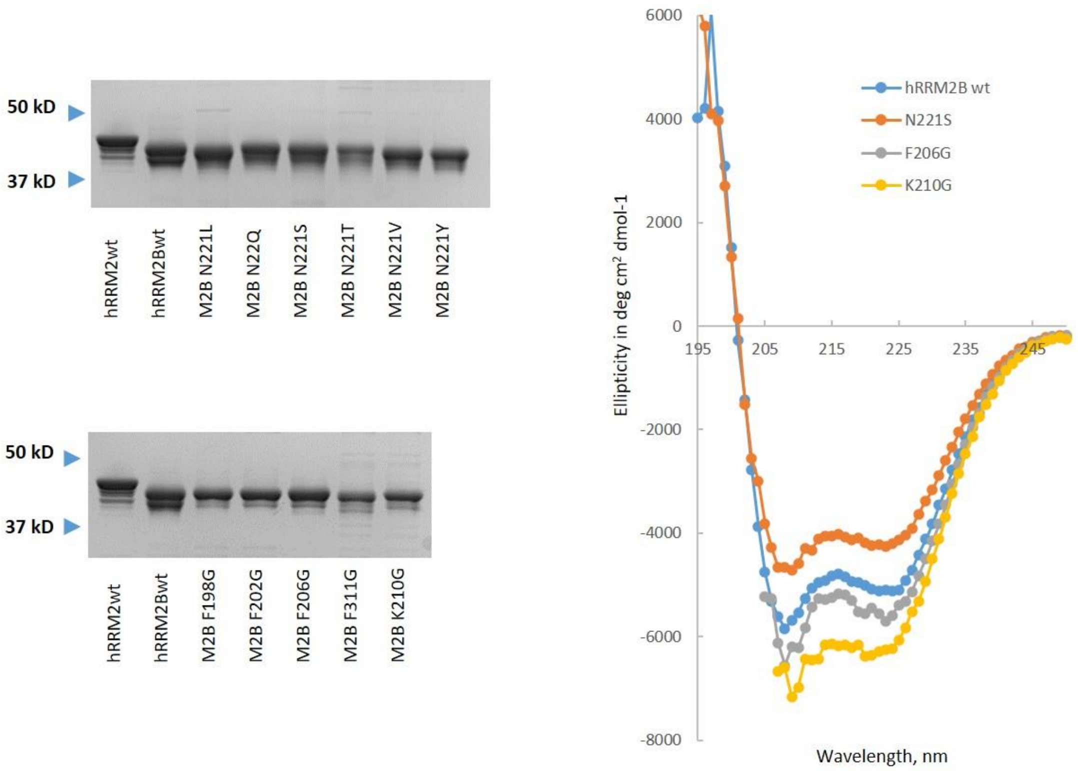

Figure S1: Protein expression and circular dichroism spectra. S1a) *In vitro* expression and purification of wildtype hRRM2 and hRRM2B, as well as hRRM2B mutants (N221L, N221Q, N221S, N221T, N221V, N221Y, F198G, F202G, F206G, F311G, K210G) shown in 4-12% Bis-Tris SDS PAGE gels. The original gels are presented in Supplementary Figure S4. S1b) CD spectra of hRRM2B wildtype and mutants N221S, F206G, and K210G. The spectra were recorded at 25℃ in 0.4 μg/μl phosphate buffer (pH7.0) with a 1 mm path length quartz cuvette in an AVIV Model 430 CD spectrometer. Each spectrum shown represents the average of three.

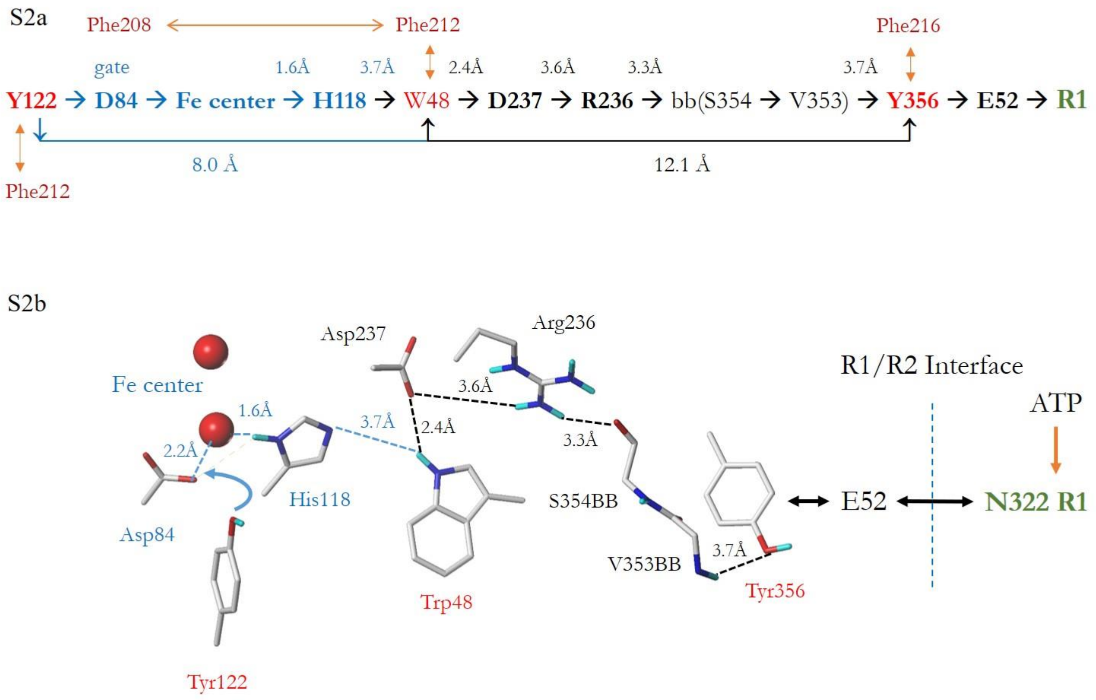

Figure S2. The proposed PCET pathway from Y122 to Y356 in *E. coli* RRM2. S2a) Y122 is the radical generation and storage site that passes radical through the D84 gate. bb refers to the backbone of the protein. W48 colored in red. The components connecting Y122 to W48 are depicted in blue, those from W48 to Y356 are in black. The residues of the phenylalanine chain involved in regulating pathway are shown along the pathway and the interactions are highlighted as two-way arrows. S2b) The possible hydrogen bonding network based on 3D structure of *E. coli* RRM2 (PDB ID 6W4X). Amino acid residues are color coded by their atom types. The numbers across the top of the pathway represent distances in angstrom (Å).

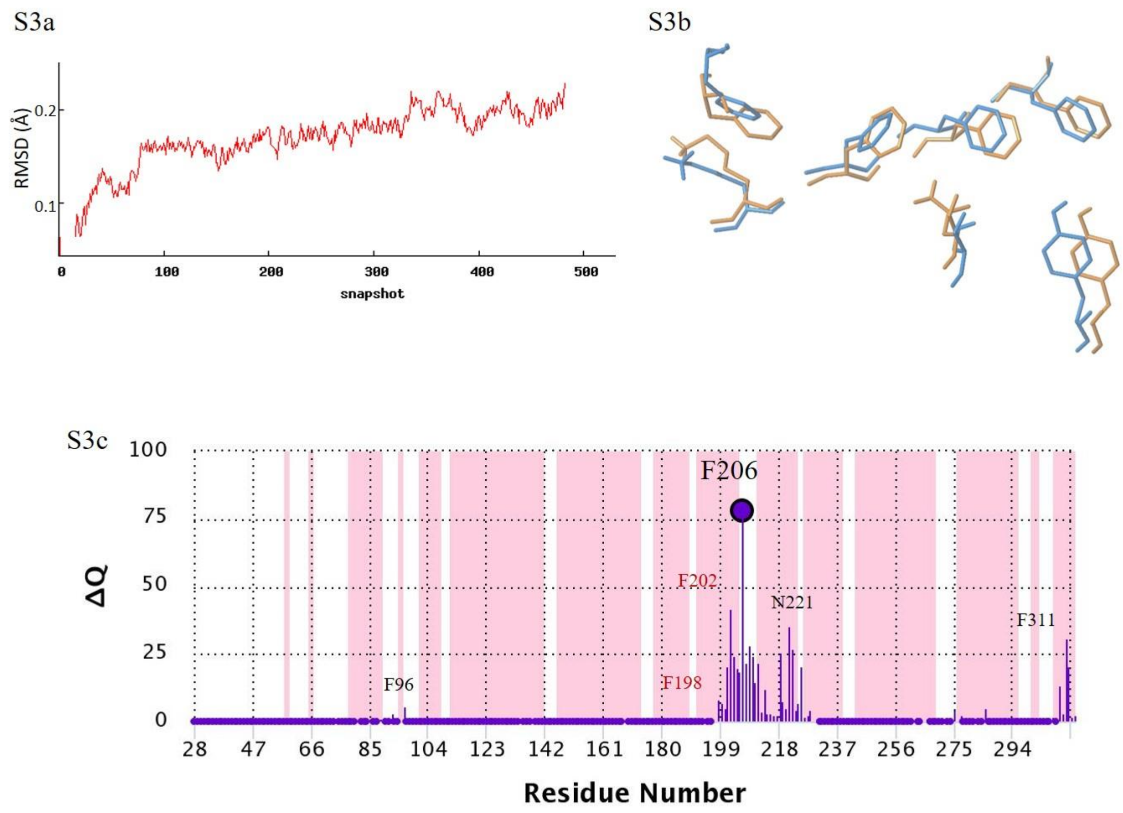

Figure S3. MD simulation and perturbation analysis of hRRM2B. S3a) RMSD values of the hRRM2B structure monitored along 500 ps MD simulation trajectory for the whole protein. S3b) Comparison of the phenylalanine chain, the anchor point residues, as well as Y138 and N221 (S221 in the mutant) in the last snapshot of hRRM2B (orange) and S221 mutant (light blue) structures retrieved from the 500 ps production phase MD simulation. The distances between N221 and F206 (black arrow), and S221 and F206 (red arrow) are shown in the figure. S3c) Perturbation profile depicts ΔQ as a function of F206 (highlighted by a circle). The shaded regions represent the secondary structure elements in the input PDB file (magenta: α-helix). The perturbed residues visualize the predicted network of F206.

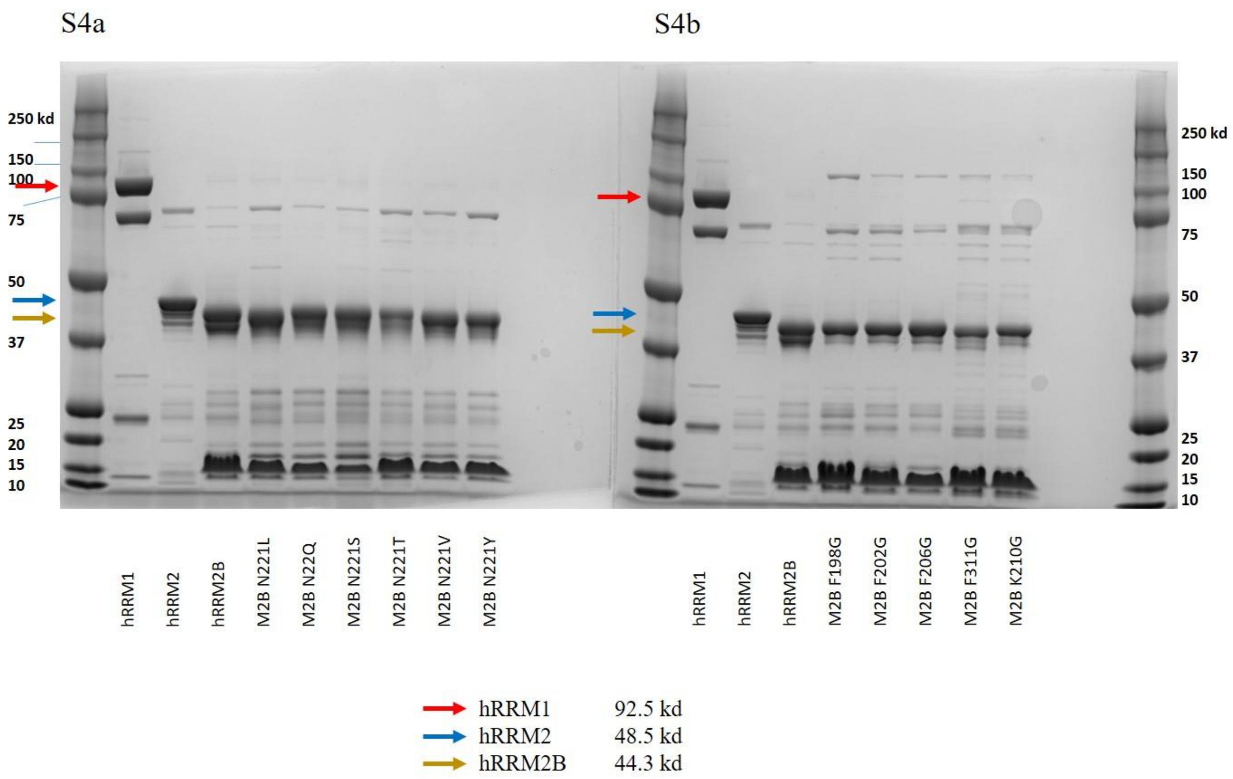

Figure S4: The original unprocessed SDS-PAGE image. In vitro expression and purification of wildtype hRRM1, hRRM2 and hRRM2B, as well as hRRM2B mutants shown in ThermoFisher 4-12% Bis-Tris SDS PAGE gels. S4a) hRRM2B N221 variants: N221L, N221Q, N221S, N221T, N221V, N221Y; S4b) hRRM2B variants: F198G, F202G, F206G, F311G, K210G). 2 ug of each protein was loaded on gel. Image acquired by BioRad Chemi Doc Touch imaging system, software version 1.1.0.4. Application setting: Coomassie Blue.

List of the primer sequences for site-directed mutagenesis on hRRM2B

hRRM2B_N221S
GGACTCACTTTTTCCAGTGAACTCATCAGCAGAG
CTCTGCTGATGAGTTCACTGGAAAAAGTGAGTCC

hRRM2B_N221T
GGACTCACTTTTTCCACTGAACTCATCAGCAGAG
CTCTGCTGATGAGTTCAGTGGAAAAAGTGAGTCC

hRRM2B_N221Y
GGACTCACTTTTTCCTATGAACTCATCAGCAGAG
CTCTGCTGATGAGTTCATAGGAAAAAGTGAGTCC

hRRM2B_N221V
GGACTCACTTTTTCCGTTGAACTCATCAGCAGAG
CTCTGCTGATGAGTTCAACGGAAAAAGTGAGTCC

hRRM2B_N221L
GGACTCACTTTTTCCCTTGAACTCATCAGCAGAG
CTCTGCTGATGAGTTCAAGGGAAAAAGTGAGTCC

hRRM2B_N221Q
GGACTCACTTTTTCCCAGGAACTCATCAGCAGAG
CTCTGCTGATGAGTTCCTGGGAAAAAGTGAGTCC

hRRM2B_F198G
GTAGAAGGAGTTTTCGGCTCAGGATCTTTTGCT
AGCAAAAGATCCTGAGCCGAAAACTCCTTCTAC

hRRM2B_F202G
TTCTTCTCAGGATCTGGTGCTGCTATATTCTGG
CCAGAATATAGCAGCACCAGATCCTGAGAAGAA

hRRM2B_F206G
GGATCTTTTGCTGCTATAGGCTGGCTAAAGAAGAGAGG
CCTCTCTTCTTTAGCCAGCCTATAGCAGCAAAAGATCC

hRRM2B_F311G
GGCAGAAAATCCTTTTGATGGTATGGAAAACATTTCTTTAGAAGG
CCTTCTAAAGAAATGTTTTCCATACCATCAAAAGGATTTTCTGCC

hRRM2B_K210G
GCTATATTCTGGCTAAAGGGGAGAGGTCTTATGCCAGG
CCTGGCATAAGACCTCTCCCCTTTAGCCAGAATATAGC
